# Effects of B-Wave Ultraviolet Supplementation Using Light-Emitting Diodes on Caged Laying Hens during the Later Phase of the Laying Cycle

**DOI:** 10.3390/ani10010015

**Published:** 2019-12-19

**Authors:** Yongxiang Wei, Weichao Zheng, Baoming Li, Qin Tong, Haipeng Shi, Xuanyang Li

**Affiliations:** 1 Department of Agricultural Structure and Environmental Engineering, College of Water Resources & Civil Engineering, China Agricultural University, Beijing 100083, China; weiyongxiang@cau.edu.cn (Y.W.); libm@cau.edu.cn (B.L.); tongqin@cau.edu.cn (Q.T.); shihaipeng@cau.edu.cn (H.S.); b20163090577@cau.edu.cn (X.L.); 2 Key Laboratory of Agricultural Engineering in Structure and Environment, Ministry of Agriculture and Rural Affairs, Beijing 100083, China; 3 Beijing Engineering Research Center on Animal Healthy Environment, Beijing 100083, China

**Keywords:** poultry, ultraviolet light-emitting diode, light supplementation, bone quality, egg quality

## Abstract

**Simple Summary:**

So far, traditional cages are still the dominant housing systems for laying hens all over the world. However, during the later phase of the laying cycle, laying hens in conventional cages are highly susceptible to osteoporosis due to a calcium deficiency, which is accompanied by a decrease in production performance and egg quality, and an increase in mortality. B-wave ultraviolet (UVB) radiation (290–315 nm) can penetrate the skin and converts 7-dehydrocholesterol (7-DHC) to previtamin D_3_, which is rapidly converted to vitamin D_3_, which promotes the body’s absorption of calcium and phosphorus, as well as bone metabolism. Hence, we hypothesize that UVB exposure using light-emitting diode (LED) lights on laying hens during the later phase of the laying cycle can promote health and welfare of layers. This study aims to improve bone quality and egg quality by utilizing light environment regulation, in order to provide a theoretical basis for the application of UVB-LED lights for laying hens during the later phase of the laying cycle.

**Abstract:**

Caged laying hens are prone to calcium deficiencies, resulting in osteoporosis and egg quality deterioration during the later phase of the laying cycle. Fluorescent light and light-emitting diodes (LEDs), which are widely used in poultry houses now, are both deficient in ultraviolet (UV) light, the lack of which is detrimental to chickens’ welfare and health. This study was conducted to investigate the effects of UVB light supplementation using LEDs on the bone traits, blood parameters, laying performance, and egg quality for caged laying hens at 68–75 weeks. In total, 120 Jingfen laying hens were randomly assigned to four different groups, with three replicates in each group (10 hens in each cage as a replicate). UVB-LED lamps installed under the feed troughs were used to provide UVB light (296–316 nm) for the birds in the three treatment groups (1 h, 2 h, and 3 h UVB supplementation per day, respectively), while the control group was not exposed to UVB-LED light. Bone traits, egg quality, and amounts of calcium (Ca), phosphorus (P), 25-hydroxyvitamin D_3_ (25(OH)D_3_), 1,25-dihydroxyvitamin D_3_ (1,25(OH)_2_D_3_), and 7-dehydrocholesterol (7-DHC) in both the serum and egg yolks were tested during the experiment. The results demonstrated that UVB-LED exposure significantly increased the bone mineral density (BMD), egg production, and yolk 1,25(OH)_2_D_3_ concentrations (*p* < 0.05), and reduced the content of serum 7-DHC (*p* < 0.05), especially in the 2 h/day group; however, it did not improve egg quality, vitamin D metabolites, or photoproducts in the serum and yolk 25(OH)_2_D_3_ concentrations (*p* > 0.05). This study concluded that UVB supplementation using LEDs had a positive effect on caged laying hens during the later phase of the laying cycle.

## 1. Introduction

Light is one of the important environmental parameters in poultry production, not only providing illumination for the birds but also influencing their bodies’ physiological functions and behavior. Currently, artificial lighting has been widely used during the full phase of the laying cycle in confined laying hen houses. However, during the later phase of the laying cycle, laying hens in conventional cages are highly susceptible to osteoporosis, due to a calcium deficiency, which is accompanied by a decrease in production performance and egg quality and an increase in mortality [1]. It has been reported that ultraviolet (UV) light exposure could effectively improve the welfare and health of the birds [2,3,4], and be proved to be helpful for osteoporosis symptoms [5,6]. However, the artificial lighting used in poultry houses now, such as incandescent, fluorescent, and light-emitting diode (LED) light, are all deficient in UV light, which has caused some welfare implications, such as sub-optimal growth, severe leg weakness, and feather pecking and cannibalism [7,8].

According to the spectrum range, UV can be divided into A-wave ultraviolet (UVA, 315–400 nm), which can induce precipitating pigments; B-wave ultraviolet (UVB, 280–315 nm), which produces erythema and can promote mineral metabolism and vitamin D formation in the body; and C-wave ultraviolet (UVC, 100–280 nm), which has strong effects on sterilization and damages cells in the body [9]. In addition, 320 nm was the most used boundary between UV-A and UV-B, rather than the 315 nm used in some other studies [10]. In addition, UVB radiation (290–315 nm), called “healthy light”, can penetrate the skin and converts 7-dehydrocholesterol (7-DHC) to previtamin D_3_, which is rapidly converted to vitamin D_3_, which promotes the body’s absorption of calcium and phosphorus, and bone metabolism [3,4,11]. Some studies have shown that poultry preferred UV-enriched environments and benefited from UV exposure [8]. There has been evidence that providing UV light (22 µW/m^2^) for 7.8 h per day could promote the growth speed of the skeleton, leg muscle weight, the skeleton quality serum calcium, phosphorus, and the growth performance of one-day-old Arbor Acres (AA) broilers [2]. Furthermore, chickens had increased body weight, bone ash, and plasma calcium (Ca), as well as decreased incidence of rickets and tibial dyschondroplasia (TD) when exposed to UV (285–365 nm) fluorescent light radiation 24 h per day, 24 h every 2 days, or 24 h every 3 days, starting with exposure on day 1 after hatching [6]. UV fluorescent light (260–400 nm) could alter the vitamin D metabolism between a high incidence of TD and a low incidence of TD in chickens [5]. Treatment of laying hens lacking vitamin D with UVB light for 1 h per day improved laying performance and egg shell quality [12]. Kühn et al. [13] exposed 26-week-old chickens to UVB (280–320 nm) lamps for 0, 15, 30, 60, 120, 180, and 300 min per day for 4 weeks, and the results showed that the contents of vitamin D_3_ and 25-hydroxyvitamin D_3_ (25(OH)D_3_) in the egg yolk increased nonlinearly in response to the increasing daily UVB exposure times.

Most studies on UV light supplementation for birds were conducted using traditional UV mercury lamps, which had short working life, high energy consumption, and serious mercury pollution [14]. These lamps also provide UV light in a wide spectral range, including not only the light helpful for vitamin D formation, but also the light with side effects like cell damage. Ultraviolet light-emitting diodes (UV-LEDs) have emerged during the past decade, and they can provide narrow-spectrum UVB light with a long working life and robustness [15]. This promising alternative raises great interest in the research on the application of UV-LEDs for UV supplementation treatment on animals and humans [16]. Many research studies have been conducted to investigate the effects of UV light supplementation, using mercury lamps on the growth, development, and behavior of laying hens during the early phase of the laying cycle, and studies have demonstrated that the supplementation good for layers [8,13,17]. We hypothesize that UVB light supplementation could improve osteoporosis and egg quality of laying hens. However, limited information is available about the effects of UVB light supplementation on osteoporosis and egg quality for laying hens, especially during the later phase of the laying cycle.

The effects of narrow-spectrum UVB light supplementation with different exposure times provided by UVB-LEDs on the bone traits, blood parameters, laying performance, and egg quality of caged laying hens during the later phase of the laying cycle was investigated in this study. The objectives of this study were (1) to use narrow-spectrum UVB light supplementation to improve osteoporosis and egg quality of laying hens; and (2) to select the appropriate exposure duration, in order to provide a theoretical basis on the application of UVB-LED lights for laying hens during the later phase of the laying cycle.

## 2. Materials and Methods

### 2.1. Animals and Experimental Treatments

**Figure 1:**
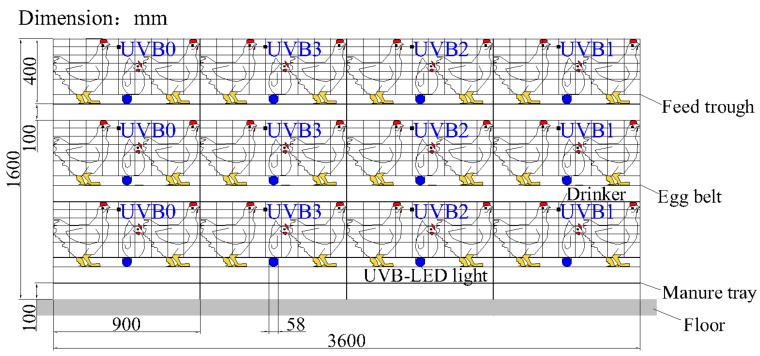
Main view of experiment cage. During the 8 weeks experiment, 1 h, 2 h, and 3 h of B-wave ultraviolet (UVB) light supplementation per day was provided for the birds in the three treatment groups (UVB1, UVB2, and UVB3, respectively), while the control group was not exposed to UVB-light-emitting diode (LED) light (UVB0).

All birds in this experiment were managed by trained staff with standing guidelines. The experiment was conducted in an environmentally controlled experimental house, in which laying hens were raised in a three-tier stacked cage system (Figure 1). In total, 120 Jingfen laying hens (Beijing Yukou Poultry Co., Ltd., Beijing, China) at an age of 68 weeks were randomly assigned to four different groups at the beginning of the experiment. Each group had three cages (0.9 m length × 0.6 m width × 0.4 m height) distributed at the three tiers of the stacked cage system, and 10 birds were raised in each cage. The stocking density was 540 cm^2^ per bird. A UVB-LED lamp (Institute of Semiconductors, Chinese Academy of Sciences, Beijing, China) was installed under the feed trough of each experimental cage in the treatment groups, facing the birds to ensure an irradiation of the legs and providing UVB light from 68 weeks to 75 weeks (8 weeks total). During the 8 weeks experiment, 1 h, 2 h, and 3 h of UVB light supplementation per day was provided for the birds in the three treatment groups (UVB1, UVB2, and UVB3, respectively), while the control group was not exposed to UVB-LED light (UVB0).

**Figure 2:**
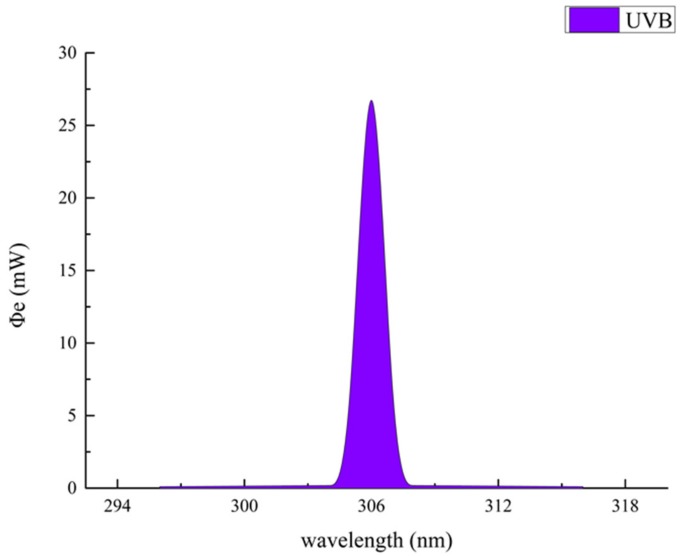
Spectrogram of ultraviolet light-emitting diode light. UVB: B-wave ultraviolet.

The emitted UVB wavelengths given by the manufacturer were in the range of 296–316 nm (Figure 2), and the emitted UVB intensity was 27 mW at a distance of 20 cm, which was measured using a spectroradiometer (HAAS-1200, Everfine Photo-E-Info Co., Ltd., Hangzhou, China). The UVB light supplementation for each treatment was evenly divided into two periods, starting at 10:00 a.m. and 15:00 p.m. A shield was installed between the adjacent individual cages to avoid unintended UVB irradiation to birds from the lamps in other cages. Each lamp’s surface was wiped with 75% alcohol twice a week to reduce the influence of dust on the light intensity.

The air temperature of the house was maintained between 16 °C and 23 °C during the experiment. Regular lighting was provided by white LEDs (15 A220 V, Natural Lighting Equipment Co., Ltd., Zhuhai, China) in this room. The lights were on at 4:30 a.m. and off at 20:30 p.m. (16L:8D), and the lighting intensity was measured by an illuminometer (SRI 2000, Shang Ze Photoelectric Co., Ltd., Hsinchu, Taiwan, China) at an average of 15 lx at the height of the birds in front of the second-tier cages. Feed with 2500 IU/kg of vitamin D_3_ and water from nipple drinkers were available ad libitum during the whole experiment.

### 2.2. Bone Collection and Tibia Traits Test

A bird randomly selected from each cage was euthanized at 0 weeks, 4 weeks, and 8 weeks of the experiment. Its left tibia was removed, and the tibia traits (bone mineral density, bone mineral content and bone area) were detected by a dual-energy X-ray bone mineral density instrument (Lunar-iDXA, GE Healthcare, Madison, Wisconsin, United States).

### 2.3. Blood Sample Collection and Analysis

Blood samples were collected in anticoagulant blood vessels from the left-wing veins of the same three randomly marked birds at the beginning of the experiment in each cage at 0 weeks, 4 weeks, and 8 weeks of the experiment. They were stored at −20 °C before being delivered to the Beijing Huaying Biotechnology Research Institute (Beijing, China) for testing on the same day. The amounts of Ca, phosphorus (P), 25-hydroxyvitamin D_3_ (25(OH)D_3_), and 1,25-dihydroxyvitamin D_3_ (1,25(OH)_2_D_3_) were determined at 0 weeks, 4 weeks and 8 weeks, and the amount of 7-dehydrocholesterol (7-DHC) was determined at 8 weeks of the experiment. The 7-DHC baseline was assumed to be the same because the same batch of laying hens were used in the experiment.

### 2.4. Egg Sample Collection and Analysis

The egg number for the hens in each group was recorded daily. The egg production is the ratio of the average number of eggs each group within a week to the total number of hens each group (30 hens each group at the beginning of experiment). Three eggs were randomly selected from each cage on the same day at 0 weeks, 4 weeks, and 8 weeks of the experiment. The weights of the egg, egg yolk, and eggshell were measured using an electronic balance (precision 0.01 g, JJ-500, G&G, Kaarst, Germany). The eggshell thickness was measured using a micrometer screw with an accuracy of 10 µm (NFN 380, FHK Co., Ltd., Tokyo, Japan). Shell fragments were collected from the equatorial area of each egg, and the inner amniotic membrane was removed. Then, the eggshell stability was determined by an electronically controlled breaking strength tester (model-II, Robotmation Co., Ltd., Tokyo, Japan). The 1,25(OH)_2_D_3_ and 25(OH)D_3_ levels in the freeze-dried egg yolks of another two randomly collected eggs from each cage were also determined by the Beijing Huaying Biotechnology Research Institute at 0 weeks, 4 weeks, and 8 weeks of the experiment. The change rate is the ratio of the data after the week to the data before the week.

### 2.5. Statistical Analysis

Data are presented as the means ± standard error (SE). Statistical analyses were performed by using linear mixed models parameterized with SPSS (IBM SPSS Statistics 22.0, New York, United States). The linear mixed model included the cage, the sample order, the sampling week, the UVB exposure time, and the interaction between the week and the UVB exposure time (week × exposure time). Effects in the statistical model were tested simultaneously, and the effects were removed from the original model when they were not significant. When the effect was statistically different (*p* < 0.05), further analysis was needed. One-way repeated measures analysis of variance (ANOVA) was applied for post-hoc group comparisons.

## 3. Results

### 3.1. Bone Traits

The bone traits of the birds in different groups are shown in Table 1. No significant change in either the bone mineral content or the area was found over the exposure duration (0, 4, and 8 weeks) between the treatment groups and the control group. However, the treatment groups and control group showed significant differences in bone mineral density (BMD) during the full trial period (*p* < 0.05). As shown in Table 1, the BMD of the birds in both the control group and the 1 h/day UVB-LED treatment group tended to decease over the duration, while that in both the 2 h/day and the 3 h/day UVB-LED treatment groups showed an increasing trend over the exposure duration. The 1 h/day UVB-LED treatment decreased the bone mineral content of the birds compared with those who were untreated and the 2 h/day UVB-LED treatment group (*p* < 0.01). Compared with the 1 h/day UVB-LED treatment group, the bone area in the control group and the 2 h/day UVB-LED treatment group increased (*p* < 0.01).

### 3.2. Vitamin D Metabolites and Photoproducts in the Serum

The vitamin D metabolites and photoproducts in the serum in the different groups are shown in Table 2. No significant changes in the contents of Ca, P, 1,25(OH)_2_D_3_, or 25(OH)D_3_ over the exposure duration (0, 4, and 8 weeks) were found in any group. However, the content of 7-DHC was significantly affected by the UVB-LED exposure (*p* < 0.01). In addition, the P content and 1,25(OH)_2_D_3_ concentration were significantly affected by the exposure duration, but not by the UVB-LED exposure (*p* < 0.05).

### 3.3. Laying Performance and Egg Quality and Vitamin D Metabolites in the Egg Yolk

**Figure 3:**
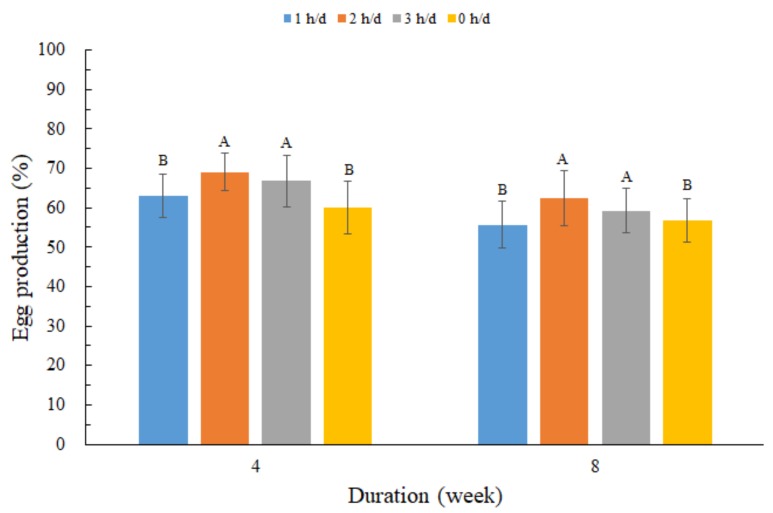
Effect of different UVB-LED light exposure durations on egg production. During the 8 weeks experiment, UVB-LED light supplementation was provided for the birds in the three treatment groups (1 h, 2 h, and 3 h per day), while the control group was not exposed to UVB-LED light. Data are presented as the means ± standard error (SE). A, B—Within a same week, different capital letters indicate significant differences (*p* < 0.05).

Figure 3 shows the egg production in each group. During the whole experiment, the number of eggs in the 2 h/day group and the 3 h/day group were significantly more than that in the control group and the 1 h/day group (*p* < 0.05). The egg quality for different groups are shown in Table 3. No significant differences were detected between treatment groups and the non-exposed group for the traits of eggshell thickness, egg shell weight, egg weight, and egg yolk weight (*p* > 0.05). However, there were significant changes in egg shell strength between the experiment group and control group over the exposure duration (0, 4, and 8 weeks) (*p* < 0.05).

**Figure 4:**
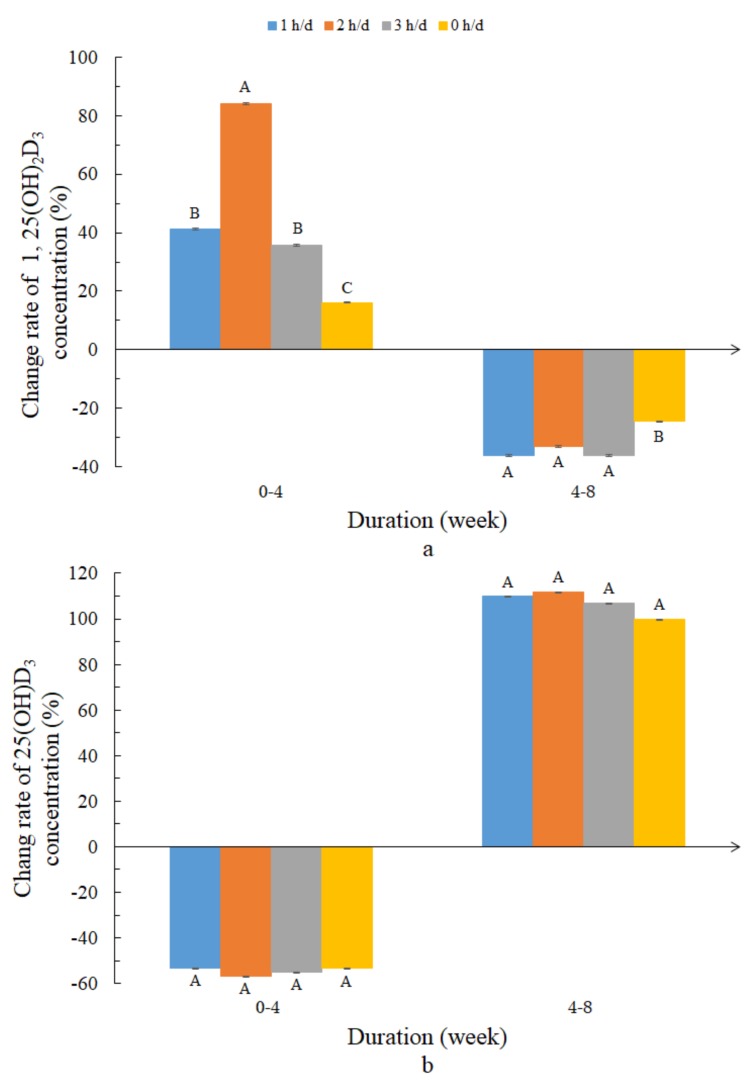
Effect of different UVB-LED light exposure durations on yolk 1,25(OH)_2_D_3_ and 25(OH)D_3_. During the 8 weeks experiment, UVB-LED light supplementation was provided for the birds in the three treatment groups (1 h, 2 h, and 3 h per day), while the control group was not exposed to UVB-LED light. The change rate of 0–4 week: the ratio of the average concentration at the 4 weeks to the average concentration at the 0 weeks; the change rate of 4–8 week: the ratio of the average concentration at the 8 weeks to the average concentration at the 4 weeks. Data are presented as the means ± standard error (SE). ^A, B, C^: Different capital letters indicate significant differences in the same duration (*p* < 0.05). (**a**) effect of different UVB-LED light exposure durations on change rate of yolk 1,25(OH)_2_D_3_ concentration; (**b**) effect of different UVB-LED light exposure durations on change rate of yolk 25(OH)D_3_ concentration

**Figure 5:**
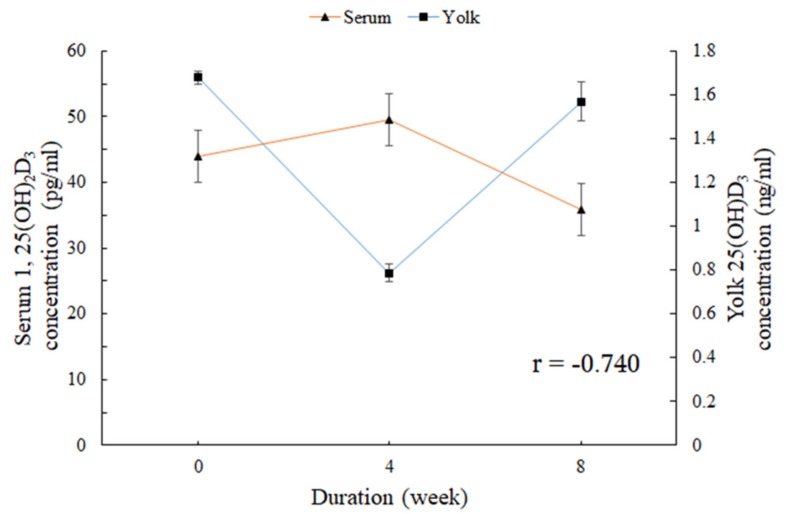
Correlation between serum 1,25(OH)_2_D_3_ concentration and yolk 25(OH)D_3_ concentration. Data are presented as the means ± standard error (SE), and *r* is the correlation coefficient.

The change rates in yolk 1,25(OH)_2_D_3_ and 25(OH)D_3_ concentrations in the different groups are shown in Figure 4. As shown in Figure 4a, for 4 weeks of UVB-LED exposure, the change rate in the yolk 1,25(OH)_2_D_3_ of the treated laying hens, especially the 2 h/day group, was higher than that of the control group. For 8 weeks of UVB-LED exposure, no significant change was found in the change rate of yolk 1,25(OH)_2_D_3_. During the whole experiment, UVB-LED treatment had an overall promoting effect on yolk 1,25(OH)_2_D_3_ concentration, especially the 2 h/day group (the rate of increase was significantly greater than the rate of decrease). No significant change in the yolk 25(OH)D_3_ concentration over the exposure duration (0, 4, and 8 weeks) was found in any group (Figure 4b). There was a negative correlation (*r* = −0.740) between serum 1,25(OH)_2_D_3_ concentration and yolk 25(OH)D_3_ concentration (Figure 5).

## 4. Discussion

This study aimed to investigate whether UVB-LED exposure was capable of improving bone quality, egg quality, and vitamin D metabolites in laying hens during the later phase of the laying cycle. The results demonstrate that UVB-LED exposure could slightly increase the bone mineral density, egg production, and yolk 1,25(OH)_2_D_3_ concentration in laying hens and reduce the content of serum 7-DHC, especially for the 2 h/day UVB-LED group; however, it did not improve the egg quality, vitamin D metabolites, and photoproducts in the serum or yolk 25(OH)_2_D_3_ concentrations. These findings clearly show that UVB-LED exposure is a key factor in improving bone quality and production performance and modulating the yolk 1,25(OH)_2_D_3_ concentration.

As age increases, the mineralized structural bone of laying hens decreases, leading to skeletal fragility and bone breakage, which has been called osteoporosis [18,19] and is an important issue for laying hens. It can result in increasing mortality and negative effects on production performance [20]. Moreover, osteoporosis is also closely related to eggshell quality [21]. The results of this study demonstrate that UVB-LED supplementation could promote the bone mineral density of laying hens during the later phase of the laying cycle. It was confirmed by a previous study that UV radiation can improve the skeleton quality (the density of skeleton mineralization) of chickens aged 0~6 weeks [2]. This is due to the bone mineral density, which is a very important measure of bone quality and is positively related to the bone quality [22]. In this study, UVB-LED supplementation might increase the percentages of calcium and phosphorus in the tibia bone, in turn affecting the bone mineral density and improving the bone quality. In addition, if the tibia bone were strengthened, UVB-LED supplementation could be utilized to meet the hen’s Ca requirement to prevent osteoporosis at the later phase of the laying cycle. With respect to the bone area and bone mineral content, the bones were not influenced by the UVB-LED supplementation. The growth plate of the laying hens most likely closed during the later phase of the laying cycle, resulting in the ceased development of the bones [23]. Since nutrition is not a direct cause of osteoporosis [24], and maximum limits for cholecalciferol supplementation in animal feeds are specified in most countries, UVB-LED supplementation can be a better choice for enhancing vitamin D to improve bone quality.

The egg production of laying hens during the later phase of the laying cycle was affected by UVB exposure, which confirmed the previous conclusion that the eggs laid by hens exposed to the radiation from a bactericidal UV lamp increased from 10% to 19% and were significantly more than a control group without ultraviolet radiation [25]. It was also proved that the increase in production was not due to vitamin D [8,25]. However, the reason for the increase in egg production remains to be seen. As the age of laying hens increases, the eggshell quality normally deteriorates [26,27]. This study showed no significant change in the traits of eggshell thickness, egg shell weight, egg weight, and egg yolk weight, which confirmed the previous findings that UVB irradiation has no impact on eggshell thickness and stability [13]. The stratum corneum of the legs of laying hens may become thick during the later phase of the laying cycle. Thus, when the supplemental time is short and UVB radiation is low, the penetration of light might not be sufficient to influence partial egg quality [12,28]. It was likely that egg quality was also influenced by dietary vitamin D and UVB exposure, and there was an interaction between these two factors. However, eggshell strength when exposed to UVB-LED light decreased, which was inconsistent with the results found of Lewis et al. [29]. This may have been a high bone index result in deterioration in shell quality [19]. In birds, the role of vitamin D_3_ in Ca and P metabolism is crucial, due to its well-documented involvement in bone development and eggshell formation in laying hens [1]. Laying hens, during the later phase of the laying cycle, use Ca from their feed to maintain the hardness of their bones and to participate in the formation of the eggshell strength. Hence, it was postulated that increasing the vitamin D_3_ content by UVB-LED exposure might not be sufficient to improve eggshell strength.

In this study, the concentrations of Ca and P in serum were not affected by the UVB exposure. However, it was reported that the serum Ca and P contents of one-day-old chickens were significantly improved by UVB exposure [2,6,8]. This may be related to the age of laying hens, as the increase in Ca absorption occurs rapidly in the early stages, and feed with an excessive vitamin D_3_ could also improve serum Ca content [2,8]. The results showed that the contents of P and Ca of laying hens exposed to UVB were synergistic, which was inconsistent with the previous research that both P and Ca showed antagonistic effects [30]. Therefore, the dynamic change in serum P and Ca contents with time under UV irradiation and the synergistic change between serum P content and Ca content need to be studied further. The results show that the relationship between the contents of 1,25(OH)_2_D_3_ and 25(OH)D_3_ in the serum was nonlinear. This confirms the previous findings that the relationship between circulating vitamin D_3_ and 25(OH)D in both groups is not linear [31]. The 25(OH)D_3_ concentration was not affected by the UVB exposure in this study, which confirmed the previous conclusion of Lietzow et al. [12]. The synthesized or absorbed vitamin D_3_ might be hydroxylated predominantly in the liver to 25(OH)D_3_, before then being released into the serum. In addition, the UVB exposure increased the bone mineral density of the tibia. One reason for this might be that there was not more 25(OH)D_3_ content to be synthesized by the UVB to release into the serum. The results of this study conflicted with previous data from studies that the content of 7-DHC was not affected by UVB irradiation [13]. UVB irradiation could reduce the content of 7-DHC [32], and with the ovulation period of the hens, the level of the 7-DHC content showed great variations [33]. Thus, the age of laying hens may affect the results of the experiment. Because leg skin becoming thick during the later phase of the laying cycle might block most of the UV rays, we hypothesized that the intensity of the UVB-LED lamp may also be a major factor. UV (250–360 nm) was an effective method of acquiring vitamin D [34]. However, the results show that the serum 1,25(OH)_2_D_3_ content was not affected by UVB exposure. In contrast, the yolk 1,25(OH)_2_D_3_ content increased upon UVB treatment. What is more, exposure of hens to artificial UVB light was an efficient strategy for increasing the vitamin D content in egg yolks [17,35]. Thus, yolk 1,25(OH)_2_D_3_ may be the ultimate product of a UVB-LED supplement. In addition to the importance of vitamin D for the development of chicks, the 25(OH)D_3_ in the egg yolk could contribute considerably to the vitamin D supply to humans, as it was proposed to possess five times greater activity than vitamin D_3_ [36]. Increasing yolk 1,25(OH)_2_D_3_ by UVB-LED exposure means increasing the vitamin D supply to humans.

Another interesting result of this study is the observed negative correlation between the serum concentrations of 1,25(OH)_2_D_3_ and 25(OH)D_3_ in egg yolks. The skin synthesis of vitamin D_3_ moved to the eggs would in turn reduce the serum content of vitamin D_3_ [37]. That could also explain the blood 25(OH)D_3_ content being in an inverse ratio relationship with the content of the 25(OH)D_3_ inside the eggs. Hence, we could make use of the relationship between the serum 1,25(OH)_2_D_3_ concentrations and 25(OH)D_3_ concentrations in egg yolks to control egg yolk vitamin D content. However, no evidence was found for the specific relationship between serum 1,25(OH)_2_D_3_ and 25(OH)D_3_ concentrations in egg yolks.

The UVB-LED lighting was placed in the lower front part of the cages to ensure an irradiation of unfeathered legs. One main reason for this is that the content of 7-DHC in the legs of laying hens is higher than that in the back and feet [38], and the vitamin D content in the feathered leg is lower than that in the unfeathered legs [13]. Keratinocytes are the predominant cell species in the epidermis that play a central and unique role in cutaneous vitamin D_3_ metabolism [32]. UV could not involve in the hypothalamic control of reproduction, essentially because of the poor penetration of intra-cranial tissues by short wave radiation [8]. Hence, this study selected the unfeathered legs of laying hens, which have a large number of keratinocytes, to increase the UVB-LED light supplementation efficiency. However, whether UVB irradiation in the vicinity of the leg skin is more efficient in improving the vitamin D content of egg yolks than UVB irradiation from the top remains to be determined.

There are two major factors that could explain the failing effect of UVB light on egg quality and vitamin D concentration in the serum and egg yolk. These factors concern the UVB dosage and the age of the laying hens. Different light intensities and photoperiods would have different effects on the performance of pullets [39,40]. Thus, the duration of the UVB-LED light supplementation time every day, the length of the light supplementation cycle, or the intensity of the UVB-LED light supplementation could affect the experimental results. In addition, the skeleton of laying hens is 95% developed by the end of the 13th week of life, and the growing period of laying hens is primarily 0~18 weeks (Growing management of commercial pullets). Thus, the next experiment will increase the UVB dosage during the rearing period of the laying hens. However, longer-term studies and a larger sample size probably will be needed to validate that possibility.

## 5. Conclusions

In conclusion, this study showed that exposing laying hens to UVB-LEDs increased the bone mineral density, egg production, and yolk 1,25(OH)_2_D_3_ concentrations, and reduced the content of serum 7-DHC, especially in the 2 h/day group. However, it did not improve the egg quality, vitamin D metabolites, or photoproducts in the serum and yolk 25(OH)_2_D_3_ concentrations. These results should stimulate further studies on the effects of exposing chickens to UVB-LED light, but further research on how the vitamin D_3_ in the serum and yolk is synthesized is needed.

## Figures and Tables

**Table 1:** Effects of different UVB-LED light exposure durations on bone traits.

Parameters	Duration (Week)	UV-LED Exposure Time			
		1 h/day	2 h/day	3 h/day	0 h/day
Bone mineral density (g/cm^2^)	0 week	0.245 ± 0.004 ^c,A^	0.230 ± 0.003 ^d,C^	0.253 ± 0.005 ^a,B^	0.248 ± 0.003 ^b,A^
	4 weeks	0.233 ± 0.002 ^c,B^	0.235 ± 0.002 ^c,B^	0.255 ± 0.003 ^a,B^	0.245 ± 0.002 ^b,B^
	8 weeks	0.233 ± 0.003 ^d,B^	0.244 ± 0.006 ^b,A^	0.264 ± 0.008 ^a,A^	0.241 ± 0.001 ^c,C^
Bone mineral content (g)	0 week	1.87 ± 0.08 ^b,A^	1.70 ± 0.03 ^c,B^	1.97 ± 0.05 ^a,A^	1.73 ± 0.06 ^c^
	4 weeks	1.66 ± 0.005 ^B^	1.65 ± 0.05 ^AB^	1.69 ± 0.06 ^B^	1.74 ± 0.03
	8 weeks	1.57 ± 0.03 ^c,C^	1.80 ± 0.04 ^b,A^	2.04 ± 0.05 ^a,A^	1.75 ± 0.02 ^b^
Bone area (cm^2^)	0 week	7.59 ± 0.20 ^a,A^	7.39 ± 0.05 ^a^	7.78 ± 0.05 ^a,A^	6.98 ± 0.26 ^b^
	4 weeks	7.12 ± 0.03 ^AB^	7.04 ± 0.23	6.75 ± 0.15 ^B^	7.08 ± 0.14
	8 weeks	6.76 ± 0.04 ^b,B^	7.38 ± 0.10 ^a^	7.49 ± 0.12 ^a,A^	7.25 ± 0.04 ^a^

During the 8 weeks experiment, UVB-LED light supplementation was provided for the birds in the three treatment groups (1 h, 2 h, and 3 h per day), while the control group was not exposed to UVB-LED light. Data are presented as the means ± standard error (SE). ^a, b, c, d^: Within a row, different lowercase letters indicate significant differences (*p* < 0.05). ^A, B, C^: Within a column, different capital letters indicate significant differences (*p* < 0.05).

**Table 2:** Effects of different UVB-LED light exposure durations on blood parameters.

Parameters	Duration (Week)	UVB-LED Exposure Time			
		1 h/d	2 h/d	3 h/d	0 h/d
P (mmol/L)	0 week	2.41 ± 0.09 ^a,B^	1.99 ± 0.02 ^b,B^	2.20 ± 0.03 ^ab,B^	2.43 ± 0.04 ^a,B^
	4 weeks	3.31 ± 0.05 ^A^	3.37 ± 0.06 ^A^	3.11 ± 0.04 ^A^	3.00 ± 0.05 ^A^
	8 weeks	2.00 ± 0.08 ^B^	1.87 ± 0.06 ^B^	1.85 ± 0.05 ^B^	2.22 ± 0.04 ^B^
Ca (mmol/L)	0 week	8.16 ± 0.07 ^a,B^	7.37 ± 0.08 ^b,B^	7.40 ± 0.17 ^b,B^	8.03 ± 0.10 ^a,A^
	4 weeks	8.91 ± 0.10 ^a,A^	8.42 ± 0.12 ^ab,A^	8.54 ± 0.11 ^ab,A^	8.14 ± 0.14 ^b,A^
	8 weeks	3.82 ± 0.01 ^C^	3.85 ± 0.03 ^C^	3.83 ± 0.04 ^C^	3.86 ± 0.03 ^B^
1,25(OH)_2_D_3_ (pg/mL)	0 week	51.54 ± 2.6 ^a,B^	30.62 ± 2.9 ^c,B^	34.21 ± 0.4 ^c,B^	43.94 ± 4.1 ^b,B^
	4 weeks	69.91 ± 1.8 ^a,A^	59.91 ± 3.5 ^c,A^	62.74 ± 3.3 ^b,A^	49.58 ± 3.8 ^d,A^
	8 weeks	35.20 ± 1.3 ^C^	35.62 ± 1.4 ^B^	35.35 ± 1.5 ^B^	35.85 ± 1.4 ^C^
25(OH)D_3_ (ng/mL)	0 week	37.11 ± 1.1	29.04 ± 1.6	33.07 ± 0.7	33.64 ± 1.9
	4 weeks	48.02 ± 1.5	40.85 ± 1.8	42.62 ± 1.7	36.23 ± 2.2
	8 weeks	21.06 ± 1.4	21.02 ± 1.4	21.05 ± 1.6	20.10 ± 1.4
7-DHC (mg/g)	8 weeks	19.00 ± 1.8 ^b^	17.25 ± 3.4 ^b^	14.20 ± 1.2 ^b^	33.50 ± 3.6 ^a^

During the 8 weeks experiment, UVB-LED light supplementation was provided for the birds in the 3 treatment groups (1 h, 2 h and 3 h per day), while the control group was not exposed to UVB-LED light. Data are presented as the means ± standard error (SE). ^a, b, c, d^: Within a row, different lowercase letters indicate significant differences (*p* < 0.05). ^A, B, C^: Within a column, different capital letters indicate significant differences (*p* < 0.05).

**Table 3:** Effect of different UVB-LED light exposure durations on egg quality.

Parameters	Duration (Week)	UV-LED Exposure Time			
		1 h/day	2 h/day	3 h/day	0 h/day
Egg shell thickness (mm)	0 week	0.28 ± 0.02	0.28 ± 0.01	0.27 ± 0.01	0.26 ± 0.02
	4 weeks	0.27 ± 0.03	0.27 ± 0.02	0.29 ± 0.02	0.30 ± 0.01
	8 weeks	0.26 ± 0.02	0.26 ± 0.01	0.28 ± 0.01	0.28 ± 0.02
Egg shell weight (g)	0 week	6.5 ± 0.37	6.8 ± 0.26	6.6 ± 0.54	6.4 ± 0.64
	4 weeks	6.4 ± 0.72	6.8 ± 0.40	6.9 ± 0.84	7.0 ± 0.35
	8 weeks	6.0 ± 0.43	6.3 ± 0.26	6.4 ± 0.63	6.7 ± 0.39
Egg weight (g)	0 week	63.9 ± 2.55	66.9 ± 4.77	64.5 ± 4.75	59.6 ± 3.75
	4 weeks	64.3 ± 3.34	68.3 ± 1.93	65.3 ± 4.65	65.0 ± 3.23
	8 weeks	60.0 ± 1.97	63.7 ± 2.14	61.1 ± 4.77	64.2 ± 1.14
Egg yolk weight (g)	0 week	16.7 ± 1.00	16.9 ± 1.03	16.7 ± 0.98	15.9 ± 1.37
	4 weeks	16.9 ± 1.01	18.5 ± 1.21	18.0 ± 1.17	17.6 ± 0.64
	8 weeks	17.8 ± 0.56	18.2 ± 0.97	17.4 ± 1.92	17.9 ± 1.07
Egg shell strength (kg/cm^3^)	0 week	2.88 ± 0.53 ^b,A^	2.77 ± 0.65 ^c,A^	3.17 ± 0.48 ^a,A^	3.10 ± 0.71 ^a,B^
	4 weeks	2.72 ± 0.64 ^c,B^	2.50 ± 0.40 ^d,B^	2.95 ± 1.08 ^b,B^	3.30 ± 0.58 ^a,A^
	8 weeks	2.70 ± 0.71 ^b,B^	2.30 ± 0.79 ^d,C^	2.56 ± 0.56 ^c,C^	3.37 ± 0.85 ^a,A^

During the 8 weeks experiment, UVB-LED light supplementation was provided for the birds in the three treatment groups (1 h, 2 h, and 3 h per day), while the control group was not exposed to UVB-LED light. Data are presented as the means ± standard error (SE). ^a, b, c, d^: Within a row, different lowercase letters indicate significant differences (*p* < 0.05). ^A, B, C^: Within a column, different capital letters indicate significant differences (*p* < 0.05).
